# The costs of wind energy permitting compliance actions for regulated bats in the US

**DOI:** 10.1371/journal.pone.0322005

**Published:** 2025-05-05

**Authors:** Christian Newman, Katie C. Surrey

**Affiliations:** Electric Power Research Institute, Palo Alto, California94304, United States of America; Institute of Geographic Sciences and Natural Resources Research Chinese Academy of Sciences, CHINA

## Abstract

The wind industry’s expansion in North America due to the need to provide clean energy is leading to increased regulatory concern for bats, particularly those that are endangered due to white-nose syndrome. The projected growth of installed wind capacity overlaps extensively with the ranges of several endangered and potentially regulated bat species. Wind energy operators in the US can comply with the Endangered Species Act (ESA) by submitting a Habitat Conservation Plan (HCP) and Incidental Take Permit (ITP) to the U.S. Fish and Wildlife Service. HCP documents include wind project overviews, estimates for incidental take (e.g., unavoidable fatalities), outline minimization and compensatory mitigation measures to avoid take, and often include estimated cost information for actions to implement the HCP/ITP. However, the lack of insight into specific cost data, combined with the lengthy ITP application process, has potentially led to the perception that ESA compliance imposes a costly regulatory burden on the private sector, deterring motivation for voluntary compliance. Resulting from the absence of routine reporting practices, it is not known how much it costs for companies to comply with ESA listings, nor is there a standardized database of compliance costs or a method for estimating them. This analysis of 25 publicly available project-specific HCPs published through 2022 establishes one approach to conceptualizing these costs and determined the median total cost for an HCP to be approximately $4.68 million (USD), with a notable discrepancy between the median costs for compensatory mitigation cost ($1.64 million) and fatality monitoring ($3.15 million). This analysis also created a general linear model that can be used to estimate potential project-specific costs, and overall provides better insight into the costs of complying with the ESA by identifying variables that might affect compliance costs, and estimating future costs for the wind industry.

## Introduction

As the wind industry has rapidly expanded to help meet the increasing demand for sustainable forms of energy generation, the need to assess the direct and indirect impacts on the environment, including habitat disturbance and mortality threats to wildlife, has corespondingly increased [1–4]. The wind industry has experienced some of the fastest growth in the energy sector and currently accounts for approximately 72% of all renewable power generation in the United States [5]. Wind turbines pose collision risks for both bird and bat species, and conservationists have raised concerns about the high rates of recorded bat fatalities on wind energy facilities [6–9]. Potential declines in the populations of some North American bat species may pose a larger conservation challenge in the future as wind energy development continues to expand [10,11]. Given the near certainty of future listings of at-risk bat species, which will cause increased regulatory implications for energy companies, it will be extremely important for the wind industry to better understand the implications of compliance options and requirements. Individual companies will need to be able to estimate the potential permitting costs more accurately as they plan and build out more wind energy, and other bat-wind stakeholders may want better understanding of the financial ramifications for the wind industry as a whole.

As of 2024, a total of nine North American bat species are federally listed as endangered or threatened species on the IUCN Red List [12]. The primary population threat to bat species of current regulatory concern is white-nose syndrome (WNS), a disease caused by an invasive fungus, that has impacted the Endangered Indiana bat (*Myotis sodalis*) and has caused the decline of species such as Northern long-eared bats (*Myotis septentrionalis*) by as much as 90% in certain locations [13]. As a result, the U.S. Fish and Wildlife Service (USFWS) uplisted the Northern long-eared bats from Threatened to Endangered in 2022 [14], and has proposed to list the tricolored bat (*Perimyotis subflavus*) as Endangered [14,15] and is considering the little brown bat (*Myotis lucifugus*) for future listing.

**Fig 1:**
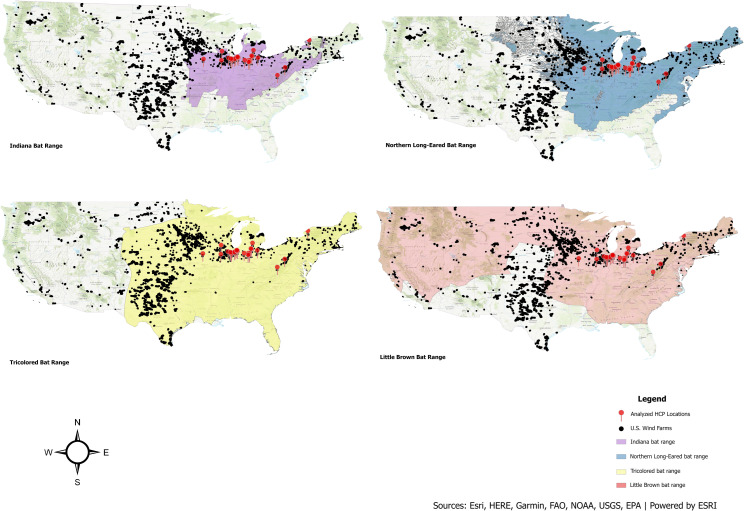
Habitat range maps and HCP locations (from this study). The colored areas identify the current ranges of four common bat species (Indiana bat, Northern long-eared bat, Tricolored bat and Little brown bat), and the locations of each of the HCPs analyzed in this study. Base map credit: ArcGIS, ESRI, Garmin, FAO, NOAA, EPA. Map and data for wind turbines are publicly available from U.S. Wind Turbine Database, provided by the U.S. Geological Survey, American Clean Power Association, and Lawrence Berkeley National Laboratory via https://energy.usgs.gov/uswtdb. Bat habitat range maps are publicly available from U.S. Fish and Wildlife Service, Department of the Interior (https://www.fws.gov/species).

Wind-related mortality is considered an additional risk factor for several of the aforementioned bat species, [16], especially as installed wind capacity is projected to grow to between 18.4 to 22.7 GW (gigawatts; [17]) by 2027 and predicted industry development already extensively overlaps the ranges of the Indiana bat and northern long-eared. Bats have been a regulatory concern for the U.S. wind industry since 2009, when the first Indiana bat was recorded as a fatality at a wind energy facility [18]. Little brown and tricolored bats have extensive ranges that cover much of the contiguous U.S. and overlap with parts of the country that will likely experience future wind development (Fig 1), so these species are also likely to face additional threats from wind turbines.

While rates of recorded bat fatalities at wind farms of species experiencing declines from WNS are relatively low, threats such as collision risks posed by wind energy can increase extinction risks to already small and declining populations. Although not currently listed under the ESA, migratory tree-roosting bats experience much higher rates of collision at wind farms. The American Wind Wildlife Institute [19] published a summary of the reported fatality incidents for wind energy development in the U.S. and found that hoary bats (*Lasiurus cinereus*), eastern red bats, (*Lasiurus borealis),* and silver-haired bats *(Lasionycteris noctivagans*) make up over 70% of the bat fatalities found on wind farms, with the hoary bat specifically comprising up to 95% of the recorded occurrences found in post construction monitoring studies across U.S. [19,20]. Some population estimates predict that hoary bat populations could experience a 50% decline by 2028 [11] without large-scale fatality reduction.

The U.S. wind industry’s compliance focus has thus far been predominantly on the Indiana bat, northern long-eared bat, tri-colored bat, and little brown bat due to their current and pending regulations, but the USFWS has added the hoary bat to its National Listing Work plan for a potential listing decision as early as 2028 which would increase overall regulatory risk for bats across the U.S. for wind energy [21]. When wind operators assess that there are fatality risks for regulated bats, they most often comply using Section 10 of the Endangered Species Act (ESA) by submitting an Incidental Take Permit (ITP) and a Habitat Conservation Plan (HCP) to the USFWS. This process is done in consultation with the USFWS and is usually contracted out to environmental consultants. An HCP characterizes the overall risk to the species, calculates “take” (i.e., the estimated amount of permitted fatality), and establishes conservation measures that will minimize and offset potential adverse effects on listed bat species. Developing an HCP offers a path for non-federal entities to comply with the ESA, and as of 2021, more than 1,300 HCPs have been approved in the U.S. across multiple species and industries [22]. The HCP also includes estimated costs for compliance activities, such as fatality monitoring and compensatory mitigation, to show how the applicant will comply with avoidance strategies agreed upon in the HCP/ITP [23].

Because of the current lack of routine reporting practices, it is not known how much it costs companies to comply with ESA listings, and there is no standardized database for ESA compliance costs. While it might seem evident that ESA compliance would influence the cost of private projects [24], there has been no formal compilation of these costs [25], nor an established method to estimate them (Surrey et al., 2020). The lack of insight into specific cost data, combined with the lengthy ITP application process, has potentially contributed to the perception that the ESA imposes a costly regulatory burden on the private sector, deterring motivation for voluntary compliance [26,27]. Better understanding of ESA compliance costs would enable companies and organizations to estimate the potential costs of incidental take more accurately, on a per-species level, and thus will be able to plan projects and understand the implications of compliance options and requirements more effectively. As bat populations continue to decline, the need to obtain ITPs will likely increase, adding further compliance requirements to companies and further necessitating HCP development and use. Overall, this paper aims to provide insight into the costs of complying with the ESA, to help identify the variables that might affect the costs of voluntary ESA compliance and therefore might help predict how future regulation changes may affect industry stakeholders.

## Methods

### HCP cost categorization method

For this study, a total of 25 HCPs were manually extracted from a publicly accessible source of the USFWS’s Environmental Conservation Online System (USFWS/ECOS, n.d.) in 2022, which was the only available data source at the time. Initially, only 13 HCPs on the online database were suitable for our analysis, however, an additional 12 HCPs were later identified, resulting in a total of 25 HCPs that could be used for general linear regression analysis (see Table 1 for the complete list). We reviewed all the HCPS, and applied a categorization protocol designed by Surrey et al., [28] that identified where mitigation costs could occur during the lifetime of a project, such as during permitting and implementation stages. Within the Implementation category, actions were further sub-categorized by:

**Table 1:** The 25 project-scale Habitat Conservation Plans included for this bat-wind energy cost analysis.

HCP Name	Primary USFWS Region	MW1	MW/Number of Turbines	Covered Bat Species	Number of Covered Bats	Total Permitted Bat Take	Year	Permit Duration2
Hog Creek	R3	66	2.2	Indiana bat; Northern long-eared bat	2	127	2020	30
Draft Crescent	R3	79.9	2.5	Indiana bat; Northern long-eared bat	1	20	2022	25
Hoopeston	R3	98	2	Indiana bat; Northern long-eared bat	2	120	2017	30
Rosewater	R3	102	1.6	Indiana bat; Northern long-eared bat	2	36	2021	6
Bluff Point	R3	120	2.11	Indiana bat; Northern long-eared bat	2	249	2020	30
Ford Ridge	R3	121	2.81	Indiana bat; Northern long-eared bat	2	18	2021	6
Bitter Ridge	R3	130	2.5	Indiana bat; Northern long-eared bat	2	114	2021	35
Pioneer Trail	R3	150	2	Indiana bat; Northern long-eared bat	1	215	2015	43
Green River	R3	194	2.62	Indiana bat; Northern long-eared bat	2	120	2022	30
Headwaters II	R3	198	4.04	Indiana bat; Northern long-eared bat	2	480	2021	30
Headwaters	R3	200	2	Indiana bat; Northern long-eared bat	2	589	2019	27
Wildcat	R3	200	2.43	Indiana bat; Northern long-eared bat	2	243	2016	28
Sugar Creek	R3	202	4.08	Indiana bat; Northern long-eared bat	2	150	2021	30
California Ridge	R3	214.4	1.6	Indiana bat; Northern long-eared bat; Little brown bat; Tricolored bat	4	155	2021	20
Indiana Crossroads	R3	302	4.19	Indiana bat; Northern long-eared bat	2	36	2022	6
Blue Creek	R3	304	2	Indiana bat; Northern long-eared bat	2	257	2020	35
Timber Road	R3	325.8	3.54	Indiana bat; Northern long-eared bat	2	340	2020	30
High Prairie	R3	400	2.29	Indiana bat; Northern long-eared bat; Little brown bat	3	186	2020	6
Jordan Creek	R3	400	3.05	Indiana bat; Northern long-eared bat	2	290	2021	30
Fowler Ridge	R3	750	1.67	Indiana bat	1	184	2013	21
Meadow Lake	R3	801	1.93	Indiana bat; Northern long-eared bat	2	894	2020	29
Copenhagen Wind	R5	70	2	Indiana bat; Northern long-eared bat	2	20	2019	25
Criterion	R5	70	2.77	Indiana bat	2	28	2014	20
North Allegheny	R5	70	2	Indiana bat	4	4	2020	25
Beech Ridge	R5	186	1.86	Indiana bat; Northern long-eared bat	2	67	2013	25

[To PE/CE: The content provided in lower font size (like hidden text) in the ManuScript. Please check and procced.]**1** Number of megawatts (MW) reported in the Habitat Conservation Plan (HCP)

[To PE/CE: The content provided in lower font size (like hidden text) in the ManuScript. Please check and procced.]**2** Length of the HCP and Incidental Take Period (ITP

- **Compensatory Mitigation** (e.g., actions to repair or restore affected habitat and species, or to compensate for unavoidable impact and take)
- **Fatality Monitoring** (e.g., activities such as vegetation clearing for monitoring plots and post-construction bat carcass surveys)
- **Administration** (e.g., administrative and overhead actions related to overall HCP implementation, such as training programs, contingency funds, and permit fees).

We isolated these different implementation costs to be used for general linear regression analysis and to calculate the average compensatory mitigation cost per bat and average total cost per megawatt generated. For the current study, we limited the data to project level (e.g., non-programmatic or large scale) HCPs within the continental U.S. that only included take of bat species and that were published and publicly accessible as of 2022 (see Table 1). There was only one multi-state (“large-scale”) HCP in our database which also included coverage for multiple species and thus did not meet our data criteria. We also identified the various project variables related to bat take, such as the number of turbines and the duration of the HCP and calculated the potential correlation between them to determine which variables might be most useful to predict project costs. The Jordan Creek and Bluff point HCPs did not explicitly list administration costs values, and Rosewater and Pioneer Trail did not have monitoring costs, so these HCPs were only included in analyses when they contained the relevant values. All analyses were performed in R (Version 1.4.1717) and Microsoft Excel 365 MSO (Version 2302).

### Cost modeling

We used the data generated from this project to develop an equation that would allow a company to estimate its potential project costs. The best-fitting model was selected using k-fold cross validation (“Leave-One-Out-Cross-Validation”), and the equation’s coefficients were generated using general linear modeling. Each coefficient included in the equation has a range of possible values, but the one selected is the most likely given the data. Because the coefficients were uncertain, this uncertainty was estimated using RMSE (root mean squared error), which was generated during model development.

**Fig 2:**
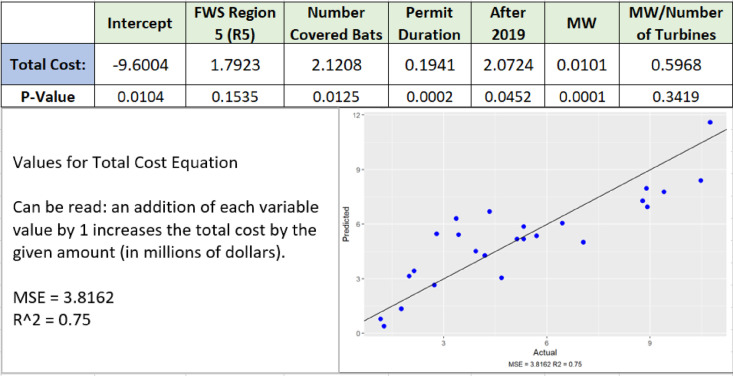
Example Output of GLM (General Linear Model) The equation can be read as “variable X adds millions of dollars to the total cost (or number of bats to total bat take) for each unit increase in the variable.” “Intercept” is a corrective term: if all variables were found to be significant, then this value would be 0. As an example, an approximate cost estimate for a 25-year project built in Region 5, after 2019, with a bat take of three (3), and 300 MW output from 100 turbines would be ~ $6,300,000 based on the following equation. $\begin{array}{cc} & \text{Total Estimated Cost}=\\ & \text{Intercept}+\text{FWS Region R5}*\text{ (1}=\text{Yes, if in Region 5, 0}=\text{No, if not in R5)}\\ & \text{+}Num Covered Bats*\text{ (Number of Bats Covered in HCP)}\\ & \text{+}Permit Duration Years*{\text{(HCP}}^{\prime }\text{s length in Years)}\\ & +\text{After}2019*\text{ (1}=\text{Yes, on or After 2019 or 0}=\text{No, before 2019)}\\ & +\text{MW}*\text{ (Expected MW)}\\ & +\text{MW/Num Turb}*\text{ (Total expected MW for project divide by number of turbines)}. \end{array}$

Based on the outputs of the applied model, an approximate total-costs equation was calculated to predict how costs will potentially increase due to changes in the variables (Fig 2). The variables (shown in Table 1) were included based on their relevance to determining the project costs.

- FWS Region: The dataset only included HCPS from Regions 3 and 5.
- MW (megawatts): Can be considered as a proxy for the size of a project.
- Number of Covered Bats: Differing amounts of bat species require different mitigation methods.
- Permit Duration: The length of a project would understandably affect overall cost.
- After 2019: Regulations and practices change over time and we found the threshold with the greatest predictive ability was 2019.
- MW/Number of Turbines: This corrective term accounts for the variation in turbine size between HCPS. Per-turbine MW output is typically reported as the amount of energy the average turbine would produce under optimal conditions (referred to as “nameplate capacity”).

## Results

### Total HCP costs

**Fig 3:**
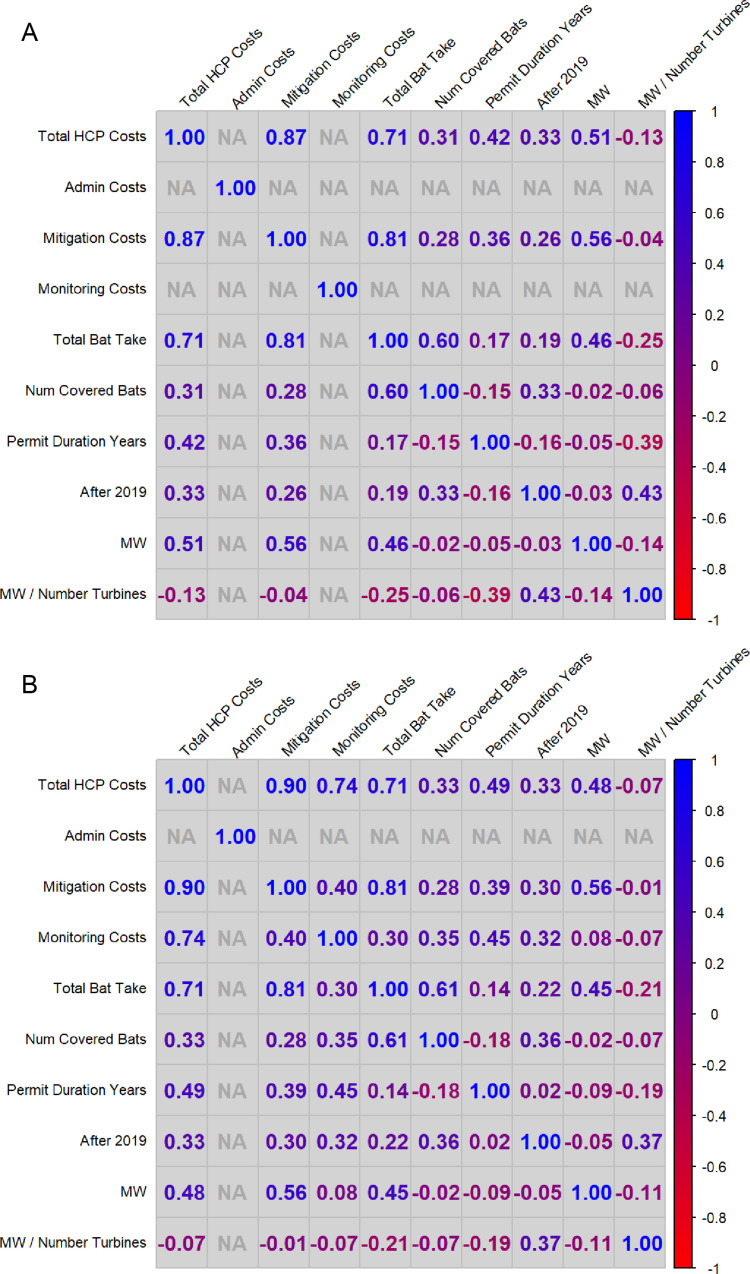
a. Correleogram of variables related to total and mitigation costs. b. Correleogram of variables related to fatality monitoring costs.

The median total cost of an HPC was $4,680,000, with a 95% CI of $3,370,000 and $6,450,000. Figs 3a and 3b show the correlograms for the variables related to Total and Mitigation Costs and Fatality Monitoring. Not all the 25 HCPs reported fatality monitoring costs (resulting in a blank column in Fig 3a), while all 23 HCPs that reported monitoring were able to be calculated (Fig 3b); therefore, the correlations from the data sets are slightly different, but the trends are similar. Overall, Total HCP Cost appeared to be highly correlated with Mitigation Costs and Total Bat Take (Fig 3a, 0.87 and 0.71, respectively), while the size of the project (e.g., MW) was not strongly correlated with total costs (0.51).

### Compensatory mitigation

**Fig 4:**
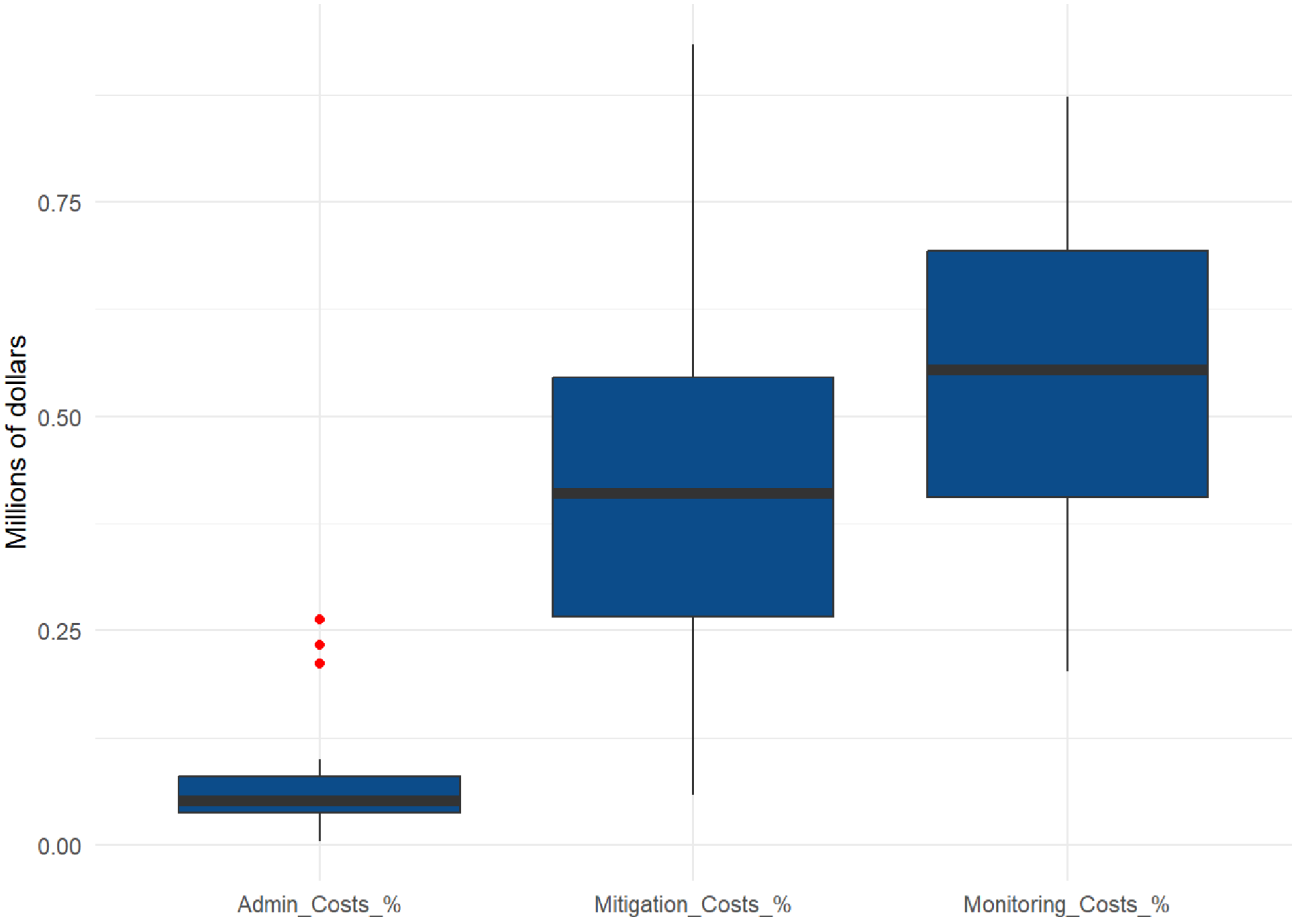
Proportions of total reported costs by category.

The median cost for compensatory mitigation was $1,640,000 with a 95% CI of $900,000 - $2,850,000 and this cost category represented approximately 40% of total HCP costs (Table 2; Fig 4). General linear regression revealed a strong positive correlation between Compensatory Mitigation costs and Total HCP Cost (0.87) and Total Bat Take (0.81) (Fig 3a), and based on our analysis, the median compensatory mitigation cost per bat was approximately $12,200 with a 95% CI ($8,470 - $15,490; Table 2). Additionally, when compensatory mitigation costs (both Total and Per MW) were compared between FWS Regions, there appeared to be a significant regional difference (p > 0.05; Table 2).

**Table 2:** Medians and confidence intervals for all variables and across all categories and between regions (R3 and R5) with highlighted variables that are significantly different. The data set of HCPs in R5 was too small to calculate median confidence interval.

Name	All Groups		R3		R5		p-value
	Median	CI (Conf.Level)	R3 Median (n = 21)	R3 CI (Conf.Level)	R5 Median (n = 4)	R5 CI (Conf.Level)	p-value R3 v R5
**Independent Variables**							
Number of Covered Bats	2	2-2	2	2-2	1.5	NA	0.1280
Total Bat Take	150	114-249	186	120-290	24	NA	0.0006
MW	194	121-214.4	200	130-304	75	NA	0.0064
# Turbines	72	49-125	74	52-134	38	NA	0.0348
MW Per # Turbines	2.2	2-2.62	2.29	2-2.81	2	NA	0.0770
**Total HCP Costs**							
Total HCP Costs	$4,680,000	$3,370,000-$6,450,000	$5,130,000	$3,370,000-$8,780,000	$3,100,000	NA	0.1100
Total per MW	$25,920	$18,670 - $30,920	$25,920	$17,350 - $32,110	$25,580	NA	0.5480
Total per Bat	$29,560	$22,750 - $36,760	$28,930	$19,260 - $36,100	$173,840	NA	0.1670
**Compensatory Mitigation Costs**							
Total Costs	$1,640,000	$900,000 - $2,850,000	$1,830,000	$1,440,000-$4,180,000	$475,000	NA	0.0001
Comp Mitigation per MW	$9,040	$6,430 - $15,570	$10,450	$7,200 - $16,930	$5,650	NA	0.0020
Comp Mitigation per Bat	$12,200	$8,470 - $15,490	$11,810	$6,940 - $14,410	$18,680	NA	0.3500
**Fatality Monitoring Costs**							
Total Costs	$3,150,000	$2,030,000-$3,320,000	$3,150,000	$2,030,000 -$3,640,000	$2,390,000	NA	0.513
Fatality Monitoring per MW	$15,720	$11,820 - $17,850	$14,830	$7,500 - $16,390	$19,350	NA	0.403
Fatality Monitoring per Bat	$16,300	$12,960 - $22,500	$16,140	$9,970 - $21,690	$124,280	NA	0.172

### Fatality monitoring costs

Fatality monitoring represented approximately 56% of the total reported costs for the HCPs analyzed (Fig 4). The median cost of fatality monitoring activities was $3,150,000 (with a 95% CI of $2,030,000- $3,320,000; Table 2) — nearly twice that of compensatory mitigation—with the median per-bat cost equaling $16,300 (with a 95% CI of $12,960-$22,500; Table 2). There was strong correlation between Fatality Monitoring and Total HCP Cost (0.74; Fig 3b) and Total Bat Take (0.71; Fig 3b); however, there were no significant regional differences (e.g., R3 vs. R5) with regards to fatality monitoring costs.

## Discussion

Overall, the data and model presented here provide a better understanding of ESA compliance costs listed in bat-wind HCPs and demonstrate at least one approach for predicting the approximate total costs of compensatory mitigation related to ESA compliance.

This research found that within wind HCPs, notably more costs on average are being directed toward fatality monitoring compared with compensatory mitigation activities for bat species, and there appeared to be significant relationships between different variables that can help predict total project costs incurred related to bat-take; specifically, mitigation cost, MW, as well as the total amount of bat take, and the number of bat species. The model produced from this research can theoretically help companies estimate costs at other U.S. mainland wind projects that plan to develop an HCP as part of the application process for an ITP to cover potential bat mortality. Ultimately this project’s results only focused on the total costs, compensatory mitigation, and fatality monitoring costs because the administrative costs were not found to strongly correlate with any of the independent variables and only represented ~5% of the total implementation costs.

While prior research found that the costs associated with compensatory mitigation represented approximately 49% of the total costs reported across HCPs [28], the compensatory mitigation costs in this project were found to only be approximately 40% of total HCP costs, despite strongly correlating to the value of the total project cost (0.87). Meanwhile, fatality monitoring costs represented over 56% of the total HCP costs, which is a notably unusual discrepancy as it would be expected that mitigation costs should be higher of the two. The high monitoring costs are likely driven by the intensity and level of effort required to conduct fatality monitoring associated with HCPs. Fatality monitoring is intended to find covered species and to ensure that the actual take levels do not exceed permitted allowances, however, because the actual occurrence of the regulated species encounters is rare (both for entire farms and on a per turbine level), the level of effort to conduct fatality monitoring can be potentially high, in order to achieve the necessary level of confidence that an encountered dead bat was actually killed by a turbine. The USFWS requires that HCP applicants use a USGS Evidence of Absence (EoA) statistical estimator [29] to produce fatality estimates when the observed number of fatalities is very low or zero during standardized carcass monitoring.Thus a high level of effort to achieve key statistical parameters or “g” value (or probability of detection of a carcass) is often needed [30]. It would be beneficial for future studies to evaluate the conservation value of high monitoring costs in relation to the funds being allocated for compensatory mitigation, and whether this discrepancy is effectively contributing towards recovery goals. It is possible that the regional cost differences in compensatory mitigation that we observed reflected differences in overall project size (e.g., MW) between the regions. For example, the medians for both project size (200 MW, n = 21) and the amount of total take (186) of Region 3 were significantly (p < 0.05) larger than those of Region 5 (75 MW, n = 4 and take = 24, respectively). Larger projects would understandably incur more costs. Additionally, the type of compensatory mitigation employed may vary by region, which would therefore affect costs. During the review of the HCPs, it was observed that all four of the Region 5 projects specifically listed “gating of known hibernacula” as their primary mitigation method. This is likely less expensive than land acquisition, which was the main form of compensatory mitigation found in the 16 HCPS from Region 3. However, it should be noted that the unequal representation of HCPs available for this analysis might also explain this discrepancy: only four HCPs were from Region 5, compared with 21 from Region 3. Additionally, this analysis only included data for two bat species, Indiana and northern long-eared. While we do not know how this model will fit for other regions or additional species such as the tricolored bat and little brown bat, it is possible that future costs for compensatory mitigation for Indiana bats and northern long-eared bats would at least be somewhat comparable within those regions.

Determining the cost estimates related to ESA compliance is challenging due to the lack of existing data that specifically relates to these actions. Companies do not typically record individual related expenses relating to planning and operations, nor do they usually make this data public or easily accessible. Along these lines, it is important to note that the omission of certain costs and actions does not indicate that these activities did not occur, nor do the costs explicitly mentioned in HCPs represent dollars actually spent (i.e., they are usually estimates). Additionally, the process of drafting an HCP is neither mandatory nor standardized, so companies and consultants will vary greatly on the level of detail they include in their plans. When cost information was included, the lack of specificity in the description often meant that the analysis depended on subjective interpretation. Because of the small sample size, this study lacked enough data to establish the statistical significance of the relationships described here. It also should be noted that there are other costs associated with HCPs, such as those associated with curtailment (defined as feathering turbines to cut in speeds or lowering the speed to other thresholds). These can result in loss of generated revenue for companies and could be quite significant depending on the level of curtailment [31].

The limited data were sufficient to construct a preliminary predictive model that could help wind energy operators predict future costs as more bat-wind HCP data become available. The model offers a quick way to see how much other HCPs have spent on similar projects without having to go through each HCP individually. A limiting factor is the dataset we used to train the model. The modeling process assumes that the 25 HCPs perfectly capture all variation seen within HCPs. So, if an HCP’s project size is substantially outside the size range of the dataset (e.g., 70–801 MW, Table 1) the model may not predict costs as effectively. This applies to all the variables, so if a company is building in a different region, this equation would probably be less accurate than for projects constructed in a region used in the model. Despite these limitations, this analysis can provide some initial insight into the variables associated with regulatory compliance, and this information can be used to estimate costs at other U.S. mainland wind projects that plan to develop an HCP as part of the application process for an ITP to cover potential bat mortality. Regression analyses suggested notable relationships between different variables that help predict total costs incurred related to bat-take; specifically, mitigation cost, megawatts, as well as the total amount of bat take, and the number of bat species.

## Conclusion

This paper is the first assessment of costs related to ESA compliance of the wind energy industry for potential bat fatalities associated with wind turbines. However, it should be noted that this paper did not attempt to estimate every cost related to ESA compliance. Companies also incur costs associated with planning, preparing, and submitting an HCP, as well as for avoiding or reducing bat fatality. Currently, curtailment (shutting off turbines during the highest periods of risk), is the primary fatality minimization method employed by wind companies [32], but which results in the loss of energy and increases the costs of wind energy annually [31,33]. While these specific costs are over and above the analysis conducted in this paper, this analysis will still help wind companies plan how they might be financially affected in the future, as more bat species become listed, and given the extensive ranges of the different endangered and at-risk bat species found across the U.S. It also provides the wind energy industry, regulators, and other bat stakeholders with an understanding of potential future cumulative bat-related wind compliance costs as more HCPs are issued to cover existing facilities as well as new facilities constructed to meet national clean energy goals.
